# Exploring spiritual health among South Korean older adults using the ICECAP-O measure: a cross-sectional study

**DOI:** 10.1186/s12955-026-02557-1

**Published:** 2026-06-09

**Authors:** Yifei Chen, Ishtiaq Ahmad, Yoshihisa Shirayama, Miyoko Okamoto, Eun Woo Nam, Motoyuki Yuasa

**Affiliations:** 1 Department of Global Health Research, Graduate School of Medicine, Juntendo University, 2-1-1, Hongo, Bunkyo-ku, Tokyo, 113-8421 Japan; 2 Department of Health Administration, Graduate School, Yonsei University, 1 Yonseidae-gil, Wonju, Gangwon-do 26493 South Korea

**Keywords:** Spiritual health, ICECAP-O, Well-being, Older people, Capability approach, South Korea

## Abstract

**Background:**

Spiritual Health (SH) is regarded as a factor that influences an individual’s overall well-being. SH is about finding meaning, having a sense of purpose, and feeling inner peace. At the same time, the holistic health viewpoints stress their proximity to human capability and the entirety of life’s quality.

**Objective:**

The present study sought to determine the relationship between capabilities and subjective well-being in older adults using the ICECAP-O (ICEpop CAPability measure for older people) instrument and also examined the role of the SH measured by PIL (Purpose in Life) as a moderator of the ICECAP-O capability outcomes.

**Methods:**

This cross-sectional study was a paper-based questionnaire survey targeting 300 older adults aged 65 and over from Wonju and Yeoju, South Korea. The demographic characteristics, health perception, ICECAP-O result and PIL score were collected. Exploratory Factor Analysis (EFA) identified latent dimensions such as HP, IL and ELS. OLS and Pearson correlation were performed on the ICECAP-O tariff, EFA factors, and PIL scores. Stata 18 was used, with a significance level set at *p* < 0.05.

**Results:**

The mean PIL score was 98.5 ± 15.6, which represents moderate well-being. Regression analyses revealed significant effects of the 3 from 5 EFA factors, including HP (β = -0.09, *p* < 0.001), IL (β = 0.03, *p* < 0.05), and ELS (β = -0.03, *p* < 0.05) on the ICECAP-O tariff. PIL scores significantly and positively correlated with ICECAP-O tariff (*r* = 0.44, *p* < 0.001), with HP showing a noteworthy moderating effect (β = 0.05, *p* < 0.01).

**Conclusion:**

The PIL score is a positive predictor of ICECAP-O among South Korea older adults, suggesting that a better sense of purpose improves capability and quality of life. Further research is needed to examine the applicability of this capability instrument across different countries and cultural contexts for spiritual health.

**Supplementary Information:**

The online version contains supplementary material available at 10.1186/s12955-026-02557-1.

## Introduction

### Advances in research on spiritual health

Spiritual Health (SH) is recognised as an important component of the overall health care system, and it has a strong impact on spiritual and physical well-being. The World Health Assembly recognised the “Spiritual Dimension” of health for the first time in Resolution 37.13 (1984) [1]. In 2004, WHO released a report titled “Promoting Mental Health” to highlight the relationship between spiritual needs and mental health [2], a connection further solidified by the 2005 Bangkok Charter, which incorporated spirituality within global health promotion [3]. In 2020, the WHO released its report Doing What Matters in Stressful Times, which accounts for both the mental and spiritual elements [4].

SH is growing to be seen as very important for life meaning and dealing with long-lasting diseases [5, 6]. It increases happiness, especially for women and the elderly, by making them stronger, more confident, and better able to help each other [7, 8]. Instruments such as the FACIT-Sp-12 are used to assess the spiritual needs of nursing homes [9]. Life satisfaction was found to be a better predictor of SH than age or health status [10]. In China, community spiritual services assisted the vulnerable elderly, and training caregivers helped with SH during COVID-19 [11, 12].

SH includes meaning, transcendence, faith, and connectedness [13]. Cultural interpretation of SH differs widely from society to society. In Islam, its focus is on divine love, duty, and the afterlife [14]; in Japan, it revolves around purposeful living through Ikigai, which involves a combination of passion, skills, contributions to society, and one’s livelihood to live a good life [15, 16]. Ikigai, meaning “A reason for being,” includes elements of passion, skills, contributing to society, and earning a living, and is considered important for well-being and SH. In healthcare, patients’ spiritual needs need to be addressed for holistic care [17]. As people get older, more spirit is needed to give meaning to being by engaging in faith and interpersonal relationships to live happier lives [18–21]. Therefore, creating a reliable method to assess non-physical health is necessary to provide personalised care and help people as they age.

### Measuring spiritual health with ICECAP-O in South Korea

South Korea is undergoing a rapid demographic transition toward a super-aged society. It is projected that by 2025, the proportion of its population aged 65 and over will exceed 20%, meeting the definition of a “super-aged” society [22]. This shift presents significant challenges to healthcare systems, economic structures, and social welfare. Older adults are increasingly affected by chronic diseases, mental health issues, and social isolation [23]. In terms of the economy, the government implemented policies such as Long-Term Care Insurance (LTCI) and pension schemes [24, 25]. While these measures provide the necessary socioeconomic support, they may not fully reflect the subjective well-being and quality of life of older adults [26].

SH is defined as a sense of meaning, purpose in their life, and connection, and is a key dimension of well-being in older adults [27]. Among its components, purpose in life (PIL) is a crucial and measurable concept, reflecting an individual’s sense of direction and fulfilment of existential meaning. SH is essential for developing culturally tuned initiatives like communal partaking endeavours [28]. Previous studies suggest that SH contributes to an improved quality of life, particularly in palliative care settings, while non-religious individuals may experience unmet spiritual needs [29]. In South Korea, influenced by Confucian traditions and urbanisation, rapid social changes and shifts in family structures have exacerbated loneliness and psychological distress among older adults [30]. These findings highlight the importance of examining measurable aspects of SH, such as PIL, in relation to broader well-being outcomes.

The ICECAP-O (ICEpop CAPability measure for Older people), based on Amartya Sen’s capability approach, assesses well-being across five domains: attachment, security, role, enjoyment, and control [31, 32]. Unlike traditional health-related quality of life measures, ICECAP is a suite of tools that can effectively capture ability levels, and it has been adapted into other versions, such as ICECAP-A, CYP, SCM, and CPM, for wider application across a broader range of ages and populations. For assessing health in older adults, it is ICECAP-O [33–35]. International validation, carried out in the UK, Europe, and Hungary, sometimes demonstrates better performance of ICECAP-O over EQ-5D-3 L for health assessment [36–40].

Although the ICECAP-O does not directly measure SH, its dimensions are conceptually related to factors such as a sense of purpose, social connection, and autonomy. Given that PIL is a core dimension of SH, examining the relationship between PIL and ICECAP-O may indirectly demonstrate the link between SH and competency-based well-being. Therefore, this study aims to explore the factors influencing ICECAP-O in older Koreans, with a focus on the role of PIL. By examining whether PIL is correlated with ICECAP-O, this study aims to clarify the relationship between SH and competency-based well-being in an ageing population.

## Methods

### Study setting and participants

This cross-sectional study included 300 participants from Wonju (Gangwon Province) and Yeoju (Gyeonggi Province), South Korea. The total population of the two cities is approximately 470,000, with about 20% of them being aged 65 and above. Therefore, the sample size reaches a 95% confidence interval.

Participants were eligible if they were aged 65 years or older, able to read and write Korean sufficiently to complete the questionnaire independently, and willing to provide written informed consent. The study excluded those with severe cognitive or psychiatric conditions affecting questionnaire completion, participants in other similar studies, and people who were in very poor health, which may have skewed the well-being or health assessments.

### Instruments

This study used paper-based questionnaires, with responses entered into an electronic database, to examine the Korean version of the ICECAP-O [32] and its relationship to individuals’ quality of life and health perceptions. Participant characteristics assessed included gender, age, educational level, family composition, income, pre-retirement occupation, religion, and engagement in social activities. Health perception was evaluated by asking participants to rate their own health and to indicate whether they felt healthier, less healthy, or about the same compared to the previous year.

The ICECAP-O measures individual capabilities based on Sen’s capability approach, in which both functionings, defined as the chosen ways of being and doing, and capabilities, understood as the freedoms to choose these ways, are essential for policy evaluation [40, 41]. PIL, regarded as a functioning, is to some extent associated with capabilities. Having a sense of purpose or meaning becomes increasingly important as people grow older. Strong links have been identified between a sense of purpose and mental and physical health, as well as longevity [42]. Furthermore, purpose explains a substantial portion of the variance in depressive symptoms, underscoring its importance in designing interventions [43].

The PIL test has 20 items that are measured on a 7-point Likert scale, with a score of 1 meaning a low sense of purpose and 7 meaning a high sense of purpose. This results in total scores ranging from 20 to 140 [44]. PIL is reliable and valid for different cultural contexts as well, validating the measure in Italian and Danish populations [45, 46]. PIL correlates substantially with subjective health; studies on heart failure and ALS patients demonstrate this, and improved quality of life and resilience are observed [47, 48]. Therefore, examining the relationship between the PIL score and the ICECAP-O instrument is necessary to assess the relevance of the ICECAP-O in measuring subjective health.

### Statistical analysis

Data analysis was conducted using Stata 18 (2023, Stata Statistical Software: TX: Stata Corp LLC) to analyse participants’ demographics, health cognition, capabilities, and well-being. Health tariffs assign utility weights to different health states and convert the ICECAP-O responses into a continuous score ranging from 0 to 1. These are needed to ensure that the research is valid [34, 38, 49, 50]. In the absence of a tariff specific to South Korea, the UK tariff was used in line with approaches taken in Germany, the Netherlands, and Hungary [37, 38]. Though this aids in comparing internationally, it may not be accurate for certain contexts due to different cultures [51, 52].

Descriptive statistics were calculated using frequencies, proportions, means, and standard deviations to summarize the characteristics of the study sample. Based on previous research, Exploratory Factor Analysis (EFA) was performed to identify latent structures in variables related to health cognition, the social experience structure, and socioeconomic status [53, 54]. Maximum likelihood estimation with varimax rotation was applied to enhance interpretability. Sampling adequacy was assessed using the KMO measure (0.63) and Bartlett’s test of sphericity (χ² = 2734.25, *p* < 0.001). The KMO value indicates an acceptable, though modest, level of sampling adequacy, suggesting that the factor structure should be interpreted with some caution. Bartlett’s test confirmed that the correlation matrix was suitable for factor analysis. Although the initial extraction using principal component analysis suggested six factors, a five-factor solution was retained due to cross-loading and interpretability considerations, accounting for 67.64% of the total variance [55]. The identified factors were labelled as Health Perception (HP), Religious Faith (RF), Marital and Family Ties (MFT), Intergenerational Living (IL), and Economic Living Status (ELS). Factor scores were standardised (mean = 0, SD = 1) and used as independent variables in subsequent analyses.

The ICECAP-O tariff is calculated using an official scoring algorithm based on the UK tariff value set. While this method is commonly used in the absence of specific country tariffs, caution is still needed when interpreting the results, as cultural differences can influence the assessment of competence status. This study used three models for multiple linear regression analysis, with the ICECAP-O tariff as the continuous dependent variable. Model 1 examined the association between factor scores (HP, RF, MFT, IL, and ELS) obtained from EFA and the ICECAP-O tariff to identify key determinants of competence-based well-being. Model 2 assessed the relationship between PIL and the ICECAP-O tariff. Although PIL was not the primary outcome variable, it was included in the model as a theoretically relevant construct representing the core dimensions of mental health and subjective well-being [56, 57]. We also calculated the Pearson correlation coefficient to further ensure the strength of the association between PIL and ICECAP-O. Model 3, as an example, further examined the moderating role of PIL in the relationship between the EFA factor HP and the ICECAP-O tariff. The model included an interaction term between HP and PIL levels (low, medium, and high) to assess whether the association between health perception and ability differs with different levels of life goals.

$$\eqalign{ & Model1:ICECAP - {0_{{\rm{Tariff}}}} \cr & = CONS\, + \,{\beta _1}F1HP\, + \,{\beta _2}F2RF\, \cr & + {\beta _3}F3MFT\, + \,{\beta _4}F4IL\, + \,{\beta _5}F5ELS \cr} $$

$$\begin{gathered} {\mathrm{Mode}}{\mkern 1mu} 2:r = \hfill \\ \frac{{\sum\nolimits_{i = 1}^N {\left( \begin{gathered} {\mathrm{PI}}{{\mathrm{L}}_i} - \hfill \\ \overline {{\mathrm{PIL}}} \hfill \\ \end{gathered} \right)\left( \begin{gathered} {\text{ICECAP - }}{{\mathrm{O}}_i} - \hfill \\ \overline {{\text{ICECAP - O}}} \hfill \\ \end{gathered} \right)} }}{{\sqrt {{{\sum\nolimits_{i = 1}^N {\left( \begin{gathered} {\mathrm{PI}}{{\mathrm{L}}_i} - \hfill \\ \overline {{\mathrm{PIL}}} \hfill \\ \end{gathered} \right)} }^2}\sum\nolimits_{i = 1}^N {{{\left( \begin{gathered} {\text{ICECAP - }}{{\mathrm{O}}_i} - \hfill \\ \overline {{\text{ICECAP - O}}} \hfill \\ \end{gathered} \right)}^2}} } }} \hfill \\ \end{gathered}$$

$$\eqalign{ & {\rm{Model}}3:ICECAP - 0Tariff \cr & = {\beta _0} + \mathop \sum \limits_{i = 1}^5 {\beta _i}{X_i} + {\beta _6}PIL \cr & + \mathop \sum \limits_{i = 1}^5 {\beta _{6 + i}}\left( {{X_i} \times PIL} \right) \cr}$$

(X_1_=F1_HP_, X_*2*_=F2_RF_, X_3_=F3_MFT_, X_4_=F4_IL_, X_5_=F5_ELS_)

## Results

### Descriptive analysis

Among 300 participants with an average age of 74 years, 109 were men, and 191 were women. Approximately 72% had at least a high school education, indicating a moderate-to-high educational level. Most participants were married (98.3%) and had children (95.0%). 66.7% of the people is religious affiliated with the highest percent of Buddhist (28.7%), Indigenous beliefs (25.4%), and Christianity (14.7%). In terms of living arrangement, 20.7% live alone, 72.0% with a spouse, 19.0% with children, 2.0% intergenerational.

Economically, 76.3% were receiving a pension (mean ± SD: 1.2 ± 0.4), and 67.9% had low financial stress (mean ± SD: 1.9 ± 1.0). Almost half were retired or not in the labour force (49.8%), and 21.4% had a temporary job. Most of the long-term occupations (37.3%) were senior or management positions. As for social participation, 71.4% of individuals participated in health or sporting activities (mean ± SD = 0.7 ± 0.5), while only 17.2% pursued hobbies. Volunteers were low at 28.8% (mean ± SD, 3.4 ± 1.0), and 25.8% were caregivers (mean ± SD, 1.7 ± 0.4).

**Fig. 1:**
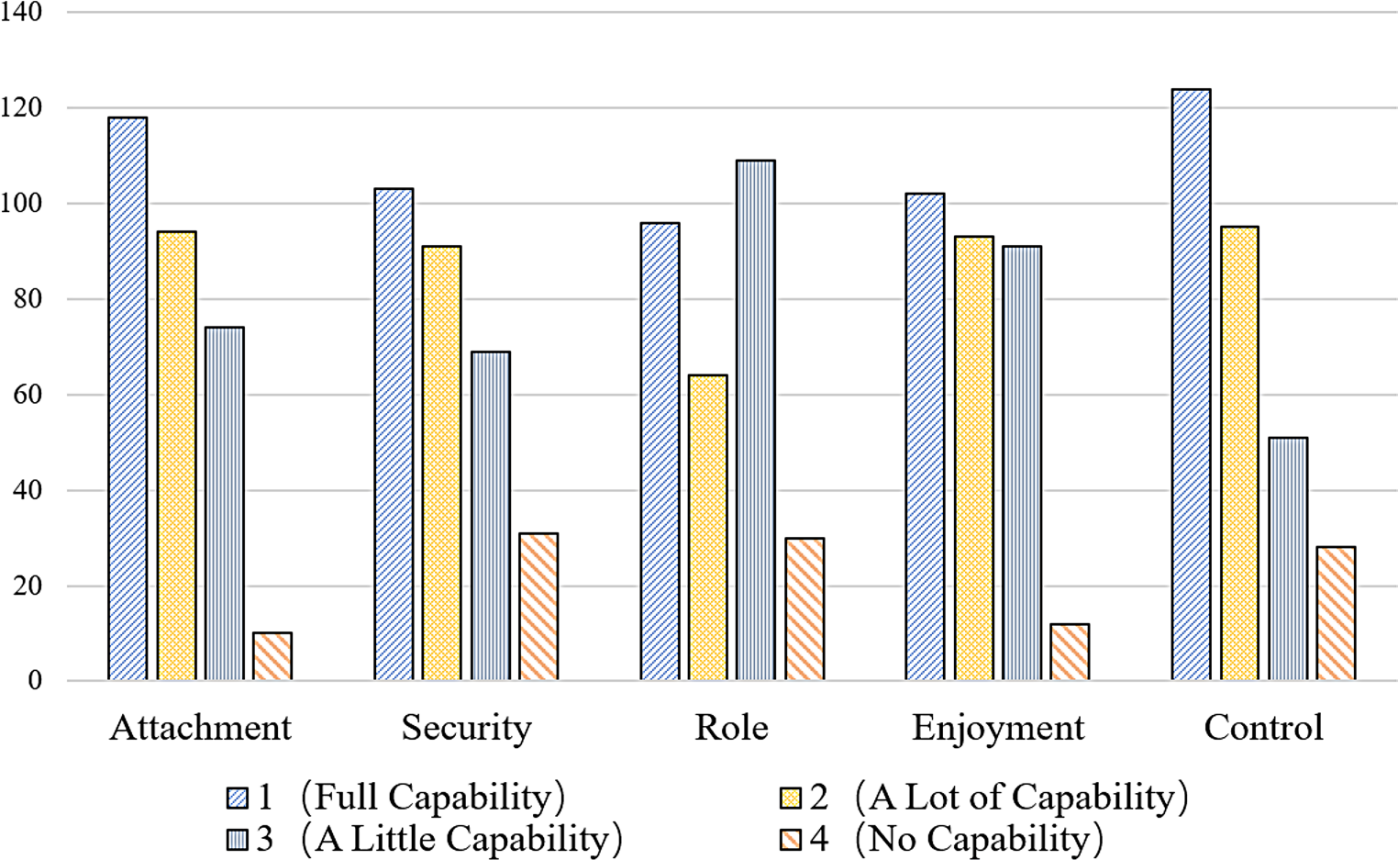
Distribution of Responses Across ICECAP-O Dimensions

Health perception was mostly good (mean ± SD: 2.4 ± 0.9), yet slightly down from last year (mean ± SD: 2.0 ± 0.9). The average PIL score was 98.5 (mean ± SD: 98.5 ± 15.6), which indicates a moderate level of well-being. Some questions had more than one answer option; percentages are based on the total number of answers given. The survey results are described more specifically, especially in the Descriptive analysis of ICECAP-O in Table 1, Supplementary Tables 1, and Fig. 1.

**Table 1:** Descriptive statistics for PIL score and ICECAP-O

	*N*	Percentage %	Mean ± SD
**ICECAP-O Attachment (love and friendship)**	296		1.92 ± 0.88
I can have all of the love and friendship that I want	118	39.9	
I can have a lot of the love and friendship that I want	94	31.8	
I can have a little of the love and friendship that I want	74	25.0	
I cannot have any of the love and friendship that I want	10	3.4	
**ICECAP-O Security (thinking about the future without concern)**	294		2.1 ± 1
I can think about the future without any concern	103	35.0	
I can think about the future with only a little concern	91	31.0	
I can only think about the future with some concern	69	23.5	
I can only think about the future with a lot of concern	31	10.5	
**ICECAP-O Role (doing things that make you feel valued)**	299		2.24 ± 1
I am able to do all of the things that make me feel valued	96	32.1	
I am able to do many of the things that make me feel valued	64	21.4	
I am able to do a few of the things that make me feel valued	109	36.5	
I am unable to do any of the things that make me feel valued	30	10.0	
**ICECAP-O Enjoyment (enjoyment and pleasure)**	298		2.04 ± 0.9
I can have all of the enjoyment and pleasure that I want	102	34.2	
I can have a lot of the enjoyment and pleasure that I want	93	31.2	
I can have a little of the enjoyment and pleasure that I want	91	30.5	
I cannot have any of the enjoyment and pleasure that I want	12	4.0	
**ICECAP-O Control (independence)**	298		1.94 ± 0.98
I am able to be completely independent	124	41.6	
I am able to be independent in many things	95	31.9	
I am able to be independent in a few things	51	17.1	
I am unable to be at all independent	28	9.4	
**PIL Score**	300		98.53 ± 15.56

### Correlation analysis results

#### Tariffs of the ICECAP-O tariff

**Fig. 2:**
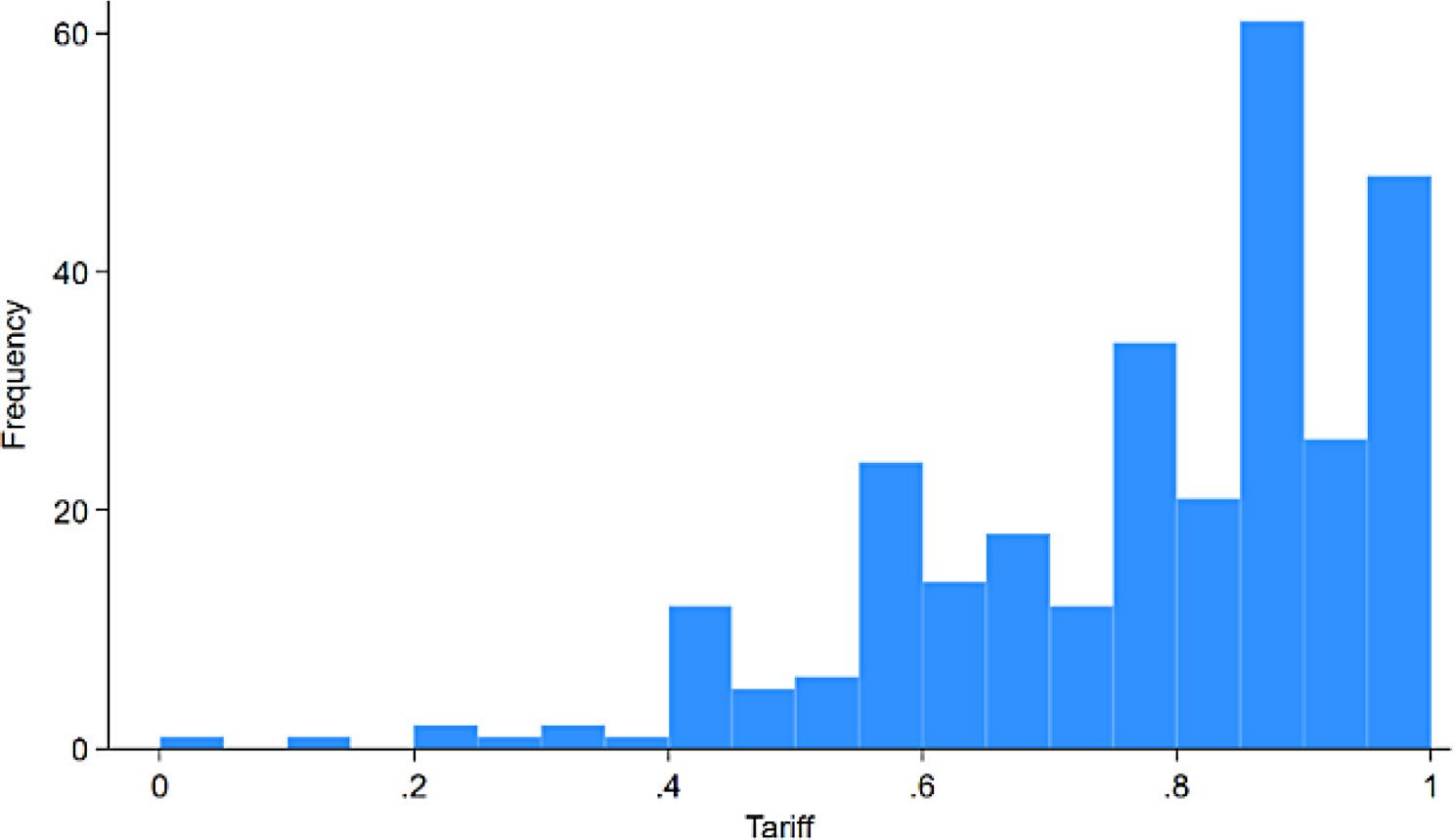
Histogram of the ICECAP-O index scores(*N* = 289)

ICECAP-O tariff rates were right-skewed; there was a high frequency of high tariffs and a low frequency of low tariffs (Fig. 2). The mean tariff rate is 0.777 ± 0.183, which is close to the average of the eight European countries and most similar to Italy and Ireland [37].

#### Exploratory factor analysis (EFA)

Table 2 gives a short description of each factor and the load factor score. HP, with self-reported health status (1.00) and comparative health status (0.64), is a subjective and relative perception of health that is closely related to SH. RF is mainly influenced by religion, primarily Indigenous Religions (0.99), which indicates that cultural identity outweighs personal religiosity. MFT indicates marital status and the structural dimension of intimate partnerships, with the highest loading for being married (0.85), living alone (0.86), and not living with a spouse (-0.94). IL represents intergenerational co-residence, primarily with children (0.60) and their spouses (0.65), with cohabitation with grandchildren (0.41) as a secondary feature, emphasising the benefits of multi-generational households for older adults. ELS loads negatively on education (-0.41) and income (-0.39) but positively on pension income (0.45), indicating that low-education, low-income individuals who rely on pensions score higher, thus representing a traditional economic characteristic factor.

**Table 2:** The EFA factors extracted from all factors based on factor loadings

Factor	Core Variables	Factor Loading	Interpretation
Health Perception (HP)	Health status Health comparison	1.00 0.64	Subjective and relative health perception
Religious Faith (RF)	Indigenous religion	0.99	Cultural identity through religious affiliation
Marital and Family Ties (MFT)	Marital status Living alone Living with spouse	0.85 0.86 -0.94	Marital status and intimate household structure
Intergenerational Living (IL)	Living with children Child’s spouse Grandchildren	0.60 0.65 0.41	Intergenerational co-residence with children and their spouses
Education and Living Standards (ELS)	Education Income Pension	-0.41 -0.39 0.45	Traditional economic dependence (low education/income, pension reliance)

### Multivariate regression analysis result

#### Regressions

The regression analysis examined the effects of multiple factors after EFA on the ICECAP-O Tariff and PIL Score (Table 3). HP showed a significant negative association with both the ICECAP-O Tariff (β = −0.09, *P* < 0.001) and the PIL (β = −0.12, *P* < 0.001), indicating that poorer perceived health is associated with reduced capability and a diminished sense of purpose. IL exhibited a positive association with the ICECAP-O Tariff (β = 0.03, *P* < 0.05) and the PIL (β = 0.08, *P* < 0.05), suggesting a beneficial contribution to well-being. MFT had a marginally negative effect on the ICECAP-O Tariff (β = -0.02, *P* > 0.05) and a significant negative effect on the PIL Score (β = -0.08, *P* < 0.05). This demonstrates the complex influence of close relationships. ELS was negatively correlated with the ICECAP-O Tariff (β = -0.03, *P* < 0.05) and was not significantly associated with the PIL score (*P* > 0.05). RF is not significantly associated with either outcome (*P* > 0.05). According to the estimation coefficients, the ICECAP-O Tariff can be represented as: ICECAP-O Tariff = 0.78 − 0.09 × HP + 0.03 × IL − 0.03 × ELS. Residuals are near zero, as expected by the model assumptions.

**Table 3:** Analysis of EFA Factors Influencing ICECAP-O Tariff and PIL Score

	ICECAP-O Tariff				PIL Score			
	Coefficient	95% CI		*P*	Coefficient	95% CI		*P*
HP	-0.09	-0.11	-0.07	0.00	-0.12	-0.18	-0.07	0.00
RF	0.01	-0.01	0.03	0.33	-0.02	-0.08	0.04	0.49
MFT	-0.02	-0.04	0.00	0.07	-0.08	-0.14	-0.02	0.01
IL	0.03	0.00	0.05	0.02	0.08	0.01	0.14	0.03
ELS	-0.03	-0.05	0.00	0.02	0.00	-0.07	0.08	0.92

**Fig. 3:**
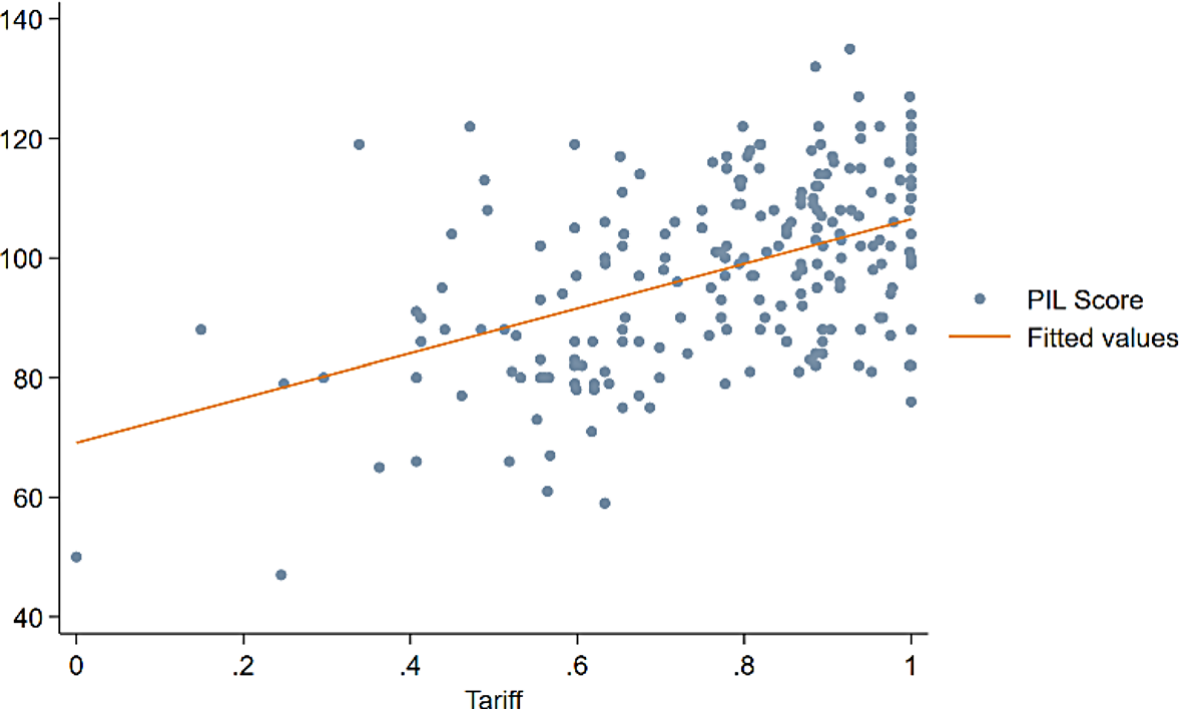
Fitted values of PIL score and ICECAP-O tariff

In the OLS regression analysis, the PIL score had a statistically significant positive relationship with the ICECAP-O tariff (β > 0, *p* < 0.001). The relationship is shown by the fitted regression line, and it is obviously going up. (Fig. 3). The Pearson correlation coefficient is always *r* = 0.44, which means that there is a moderate positive correlation and supports the results of the regression [58].

**Fig. 4:**
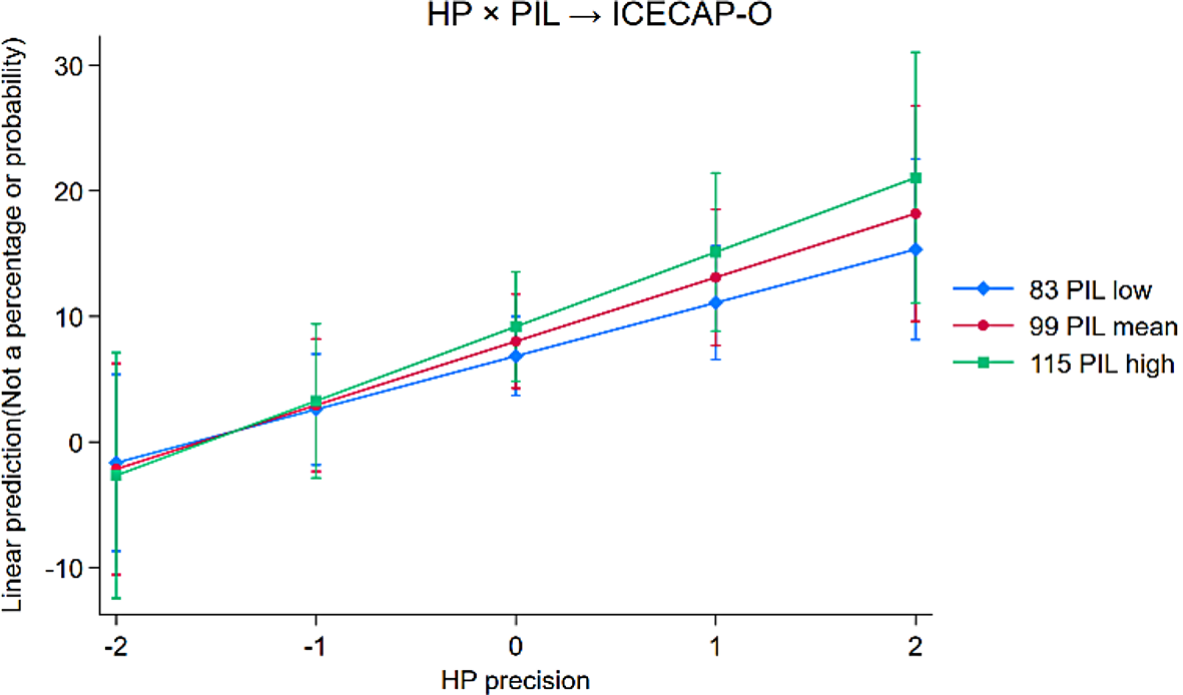
Visualization of the moderating effect of PIL on the relationship between HP and ICECAP-O

The notion of a mediating effect turns out to be a really important part of looking into health, as to why some specific things can change the way certain health factors lead to different results [59]. Table 4 shows the regression analysis of how the PIL score moderates the ICECAP-O tariff. PIL Score is a substantial positive predictor of ICECAP-O (*p* < 0.001) - a better sense of purpose improves well-being. It moderates the link between HP and ICECAP-O (*p* < 0.01), which implies that it can lessen the detrimental effects of poor health perception. RF, the PIL Score has a significant negative moderating effect (*p* < 0.05), which indicates a substitution effect. It also moderately modifies MFT(*p* < 0.05), indicating a protective role in the family context. The moderation effect for IL is nearly significant, while it is not significant for ELS. Overall, these results highlight the complex moderating role of life purpose, especially based on the significant impact of health perception on ability. Figure 4 illustrates how the intercept between HP and ICECAP-O varies with the moderating effect of PIL scores as an example of these EFA factors.

**Table 4:** Moderating effects of PIL score on ICECAP-O tariff

	ICECAP-O Tariff			
	Coefficient	95% CI		*p*
PIL Score	0.074	0.036	0.113	0
HP× PIL Score	0.052	0.014	0.091	0.008
RF× PIL Score	-0.042	-0.079	-0.006	0.023
MFT× PIL Score	0.045	0.005	0.084	0.026
IL × PIL Score	0.042	-0.001	0.085	0.053
ELS × PIL Score	0.001	-0.044	0.047	0.952

#### Fit check

Supplementary Fig. 1 shows the Q-Q plots comparing the standardised residuals’ empirical quantiles with the theoretical quantiles of the standard normal distribution. Most data points align closely with the diagonal line, indicating approximately normal residuals [60]. Supplementary Figs. 2 and 3 show similar results for the other models, confirming a good model fit and supporting the normality assumption of the residuals. Supplementary Table 2 compares the Akaike Information Criterion (AIC) and Bayesian Information Criterion (BIC) for three models [61, 62]. Model 3, with the best log-likelihood (136.434), outperforms Model 1 (119.695) and Model 2 (113.449) and has the lowest AIC (-248.8684) and BIC (-205.5109), indicating the best fit. However, its complexity may lead to overfitting. Therefore, a cross-validation method was used to evaluate the model fit.

## Discussion

Since 2006, the ICECAP-O has attracted growing interest because of its strong psychometric qualities and usefulness in economic evaluations. Nevertheless, its use in health-related research, particularly in non-Western settings, remains relatively scarce [59]. This study adds to the existing literature by investigating how SH (assessed using the PIL) influences competence-based well-being among older adults in Korea. Descriptive findings show that the study sample is characterised by relatively stable socioeconomic and family circumstances, including high rates of marriage, co-residence with family members, and attainment of secondary or higher education (Supplementary Table 1, Fig. 1). Responses to the ICECAP-O tariff are predominantly clustered in the moderate to high capability range, which is in line with previous research [63–67] and supports the reliability of the measure in this population. In addition, the mean ICECAP-O tariff score (0.777 ± 0.183) closely aligns with findings reported in studies of European populations [37, 68, 69]. The right-skewed distribution of scores (Fig. 2) further indicates that most respondents reported high ability levels, supporting the tariff’s cross-contextual applicability despite using a UK-based values set. EFA identified five explainable factors covering key dimensions of older adults’ life experience (Table 2). Among these, HP, IL, and ELS were significant predictors of the ICECAP-O tariff (Table 3). Consistent with previous research, poorer perceived health was associated with lower competence, highlighting the central role of subjective health in shaping overall well-being [70, 71, 72, 77]. ELS was also negatively correlated with competence, indicating that structural factors limit an individual’s ability to achieve their ideal life. Notably, IL was positively correlated with the ICECAP-O tariff, suggesting that living and interacting with younger family members may enhance older adults’ well-being. This finding aligns with the importance of family support in Korean culture and suggests that continued social interaction in multigenerational households may help maintain cognitive and emotional functioning [73, 74].

The second model revealed a clear positive relationship between PIL and ICECAP-O, as shown in Fig. 3, reinforcing the hypothesis that PIL is closely associated with capability-based well-being. PIL may function as a key psychological resource, strengthening individuals’ sense of autonomy, role fulfilment, and overall life satisfaction. These results offer indirect support for the idea that capability measures like ICECAP-O can capture elements of SH. Notably, the final model examining moderating effects shows that PIL shapes the relationship between perceived health and competence (Table 4; Fig. 4). The strength of the association between perceived health and ICECAP-O changes depending on the level of PIL, such that people with higher PIL exhibit a weaker negative link between perceived health and competence. This pattern may indicate a “substitution effect” between different sources of meaning. In South Korea, traditional foundations of meaning—such as family roles and Confucian religious values—are increasingly challenged by rapid urbanisation and evolving family structures. Consequently, older adults may turn more toward internal sources of meaning, such as personal life goals, especially when external support diminishes. For individuals with strong religious faith, however, meaning may stem primarily from spirituality and involvement in religious communities, thereby lessening the relative influence of personal meaning in life. This implies that different facets of SH may partly substitute for one another in sustaining well-being. This mechanism underscores the importance of culturally embedded pathways through which older adults derive meaning and preserve their sense of competence in later life.

Q-Q plots and AIC and BIC (Supplementary Figs. 1–3, and Supplementary Table 2) showed that the model fits well, and the PIL moderation model has the best validity. This study has some limitations. While the factors of ICECAP-O are complementary to the PIL scale, research on the factor structure of the five dimensions of ICECAP-O is limited [75]. Using the UK-based tariff scoring system may not fully reflect Korean cultural values; therefore, the results should be interpreted with caution. Furthermore, the relatively low KMO value suggests that while the factor structure is acceptable, it may not be robust enough. Although the PIL scale is widely used, it is still under development as a psychometric tool [76, 77]. Future research should focus on developing a culturally specific South Korea ICECAP-O tariff scoring system, conducting more rigorous factor validation, and incorporating multiple SH measurement methods to better capture the multidimensional characteristics of well-being in older adults.

## Conclusion

The results indicate that subjective health perception, socioeconomic status, and intergenerational living are important factors influencing competence-based SH. PIL was positively correlated with the ICECAP-O tariff and moderated the relationship between perceived health and competence, potentially interacting with both socioeconomic status and intergenerational cohabitation. This suggests that SH can buffer the negative impacts of poor health.

These results highlight the importance of incorporating psychological and SH dimensions into ageing assessments. Interventions that enhance a sense of PIL, such as community and social participation programs, may contribute to improving the SH of older adults. Furthermore, the findings support the use of broader competence-based measurements, such as ICECAP-O, in policy and care assessments, in addition to traditional health indicators. Future research could employ longitudinal study designs and measures of Korean cultural adaptation to further validate these findings.

## Supplementary Information

Below is the link to the electronic supplementary material.

Supplementary Material 1

Supplementary Material 2

## Data Availability

Data will be made available on request.
