# Supplementary material for: Exploring spiritual health among South Korean older adults using the ICECAP-O measure: a cross-sectional study
Source: Health Qual Life Outcomes. 2026 Jun 9;24:104. doi: 10.1186/s12955-026-02557-1 (PMC13474915; doi:10.1186/s12955-026-02557-1)
Supplement: Supplementary file 2 [file 12955_2026_2557_MOESM2_ESM.docx]

**Supplementary**

**Table 1 Demographics characteristics of the study participants**

|  | **N** | **Percentage%** |
| --- | --- | --- |
| **Gender** | 300 |  |
| male | 109 | 36.3 |
| female | 191 | 63.7 |
| **Age (Mean ± SD)** | 73.8 ± 6.5 |  |
| **Education** | 297 |  |
| Elementary school/middle school | 83 | 28.0 |
| High school | 105 | 35.4 |
| Junior college/vocational school/vocational school | 34 | 11.5 |
| University | 49 | 16.5 |
| Graduate school | 16 | 5.4 |
| Other | 10 | 3.4 |
| **Marital Status** | 299 |  |
| Never married | 5 | 1.7 |
| Currently, have a spouse or partner | 221 | 73.9 |
| Divorced from spouse or partner | 11 | 3.7 |
| Spouse or partner died | 61 | 20.4 |
| Other | 1 | 0.3 |
| **Children** | 299 |  |
| Have children (living together only) | 45 | 15.1 |
| Have children (living separately only) | 170 | 56.9 |
| Have children (living together or living separately) | 66 | 22.1 |
| No children | 15 | 5.0 |
| Other | 3 | 1.0 |
| **Cohabitor** | 378 |  |
| Alone | 62 | 20.7 |
| Spouse | 216 | 72.0 |
| Parents | 3 | 1.0 |
| Children | 57 | 19.0 |
| Spouses of children | 30 | 10.0 |
| Grandchildren | 6 | 2.0 |
| Brothers and sisters | 3 | 1.0 |
| Others | 1 | 0.3 |
| **Current Career** | 299 |  |
| Self-employed agricultural, forestry, and fishery workers (including family workers) | 46 | 15.4 |
| Self-employed commerce, industry, and service workers (including family workers) | 17 | 5.7 |
| Executives of companies or organizations | 6 | 2.0 |
| Full-time wage workers | 14 | 4.7 |
| Part-time/temporary wage workers | 64 | 21.4 |
| No work that involves income | 149 | 49.8 |
| Others | 3 | 1.0 |
| **Long Working Career** | 295 |  |
| Self-employed agricultural, forestry, and fishery workers (including family workers) | 53 | 18.0 |
| Self-employed commerce, industry, and service workers (including family workers) | 48 | 16.3 |
| Executives of companies or organizations | 110 | 37.3 |
| Full-time wage workers | 22 | 7.5 |
| Part-time/temporary wage workers | 22 | 7.5 |
| No work that involves income | 38 | 12.9 |
| Others | 2 | 0.7 |
| **Economic Status** | 296 |  |
| Living without any worries because the household is well-off | 68 | 23.0 |
| Living without much worries, although the household is not well-off | 133 | 44.9 |
| Somewhat worried because the household is not well-off | 85 | 28.7 |
| Very worried because the household is struggling | 10 | 3.4 |
| **Household Income** | 295 |  |
| Less than 2 million won | 122 | 41.4 |
| 2 million won to less than 4 million won | 96 | 32.5 |
| 4 million won to less than 6 million won | 54 | 18.3 |
| 6 million won or more | 23 | 7.8 |
| **Pension** | 300 |  |
| Yes | 225 | 75.0 |
| No | 70 | 23.3 |
| Unknown | 5 | 0.0 |
| **Caregiver** | 300 |  |
| Yes | 76 | 25.0 |
| No | 219 | 73.0 |
| Unknown | 5 | 0.0 |
| **Strong Religious Beliefs** | 297 |  |
| Very strongly | 76 | 25.6 |
| Somewhat | 122 | 41.1 |
| Not | 58 | 19.5 |
| Not sure | 41 | 13.8 |
| **Types of Religious** | 211 |  |
| Buddhism | 76 | 25.4 |
| Indigenous | 86 | 28.7 |
| Christianity | 44 | 14.7 |
| Taoism | 0 | 0.0 |
| Other | 5 | 1.7 |
| **Social Activities** | 423 |  |
| Health Sports | 212 | 71.4 |
| Hobbies | 51 | 17.2 |
| Local Events | 39 | 13.1 |
| Improving the living environment | 30 | 10.1 |
| Production Employment | 17 | 5.7 |
| Safety management | 10 | 3.4 |
| Education cultural activities | 24 | 8.1 |
| Other | 18 | 6.1 |
| No activities | 22 | 7.4 |
| **Volunteer Activity** | 291 |  |
| Support for the elderly (housework support, transportation support, etc.) | 38 | 13.1 |
| Support for childcare (childcare assistance, etc.) | 7 | 2.4 |
| Other | 37 | 12.7 |
| None | 209 | 71.8 |
| **Health Status** | 297 |  |
| Very healthy | 45 | 15.2 |
| Fairly healthy | 119 | 40.1 |
| Not very healthy | 99 | 33.3 |
| Not healthy | 34 | 11.5 |
| **Health Comparison** | 297 |  |
| Yes, healthy | 123 | 41.4 |
| Not sure | 55 | 18.5 |
| No, not healthy | 119 | 40.1 |

**Figure 5.1 Normal Q-Q plot of Model 1**

**Figure 5.2 Normal Q-Q plot of Model 2**

**Figure 5.3 Normal Q-Q plot of Model 3**

| Model | AIC | BIC |
| --- | --- | --- |
| Model 1 | -227.39 | -205.711 |
| Model 2 | -222.898 | -215.565 |
| Model 3 | -248.868 | -205.511 |

**Table 6 Model Fit Statistics: AIC and BIC for Three Models**
